# The complete nucleotide sequence of the genome of Barley yellow dwarf virus-RMV reveals it to be a new *Polerovirus* distantly related to other yellow dwarf viruses

**DOI:** 10.3389/fmicb.2013.00205

**Published:** 2013-07-23

**Authors:** Elizabeth N. Krueger, Randy J. Beckett, Stewart M. Gray, W. Allen Miller

**Affiliations:** ^1^Plant Pathology and Microbiology Department, Iowa State UniversityAmes, IA, USA; ^2^USDA/ARS and Plant Pathology Department, Cornell UniversityIthaca, NY, USA

**Keywords:** Luteoviridae phylogenetics, P0, maize yellow dwarf virus, maize virus, rhopalosiphum maidis, luteovirid

## Abstract

The yellow dwarf viruses (YDVs) of the *Luteoviridae* family represent the most widespread group of cereal viruses worldwide. They include the Barley yellow dwarf viruses (BYDVs) of genus *Luteovirus*, the Cereal yellow dwarf viruses (CYDVs) and Wheat yellow dwarf virus (WYDV) of genus *Polerovirus*. All of these viruses are obligately aphid transmitted and phloem-limited. The first described YDVs (initially all called BYDV) were classified by their most efficient vector. One of these viruses, BYDV-RMV, is transmitted most efficiently by the corn leaf aphid, *Rhopalosiphum maidis*. Here we report the complete 5612 nucleotide sequence of the genomic RNA of a Montana isolate of BYDV-RMV (isolate RMV MTFE87, Genbank accession no. KC921392). The sequence revealed that BYDV-RMV is a polerovirus, but it is quite distantly related to the CYDVs or WYDV, which are very closely related to each other. Nor is BYDV-RMV closely related to any other particular polerovirus. Depending on the gene that is compared, different poleroviruses (none of them a YDV) share the most sequence similarity to BYDV-RMV. Because of its distant relationship to other YDVs, and because it commonly infects maize via its vector, *R. maidis*, we propose that BYDV-RMV be renamed Maize yellow dwarf virus-RMV (MYDV-RMV).

## Introduction

Barley yellow dwarf viruses (BYDVs), Wheat yellow dwarf virus (WYDV), and Cereal yellow dwarf viruses (CYDVs), collectively known as yellow dwarf viruses (YDVs) are among the most economically important causal agents of disease in cereal crops. YDVs have been reported in both agriculturally important cereal crops and non-crop grasses throughout the world (El-Muadhidi et al., 2001; Hawkes and Jones, 2005; Hesler et al., 2005; Kumari et al., 2006; Power et al., 2011; Siddiqui et al., 2012; Jarošová et al., 2013). YDVs vectored primarily by the bird cherry-oat aphid, *Rhopalosiphum padi*, can cause yield reductions of 15 to 25% in barley, wheat and oat (Lister and Ranieri, 1995). The presence of BYDV correlated with a reduction in yield of winter wheat (Banks et al., 1995) as well as wheat and oats (McKirdy et al., 2002). Perry et al. (Perry et al., 2000) found an average of 30% yield loss in affected winter wheat fields. Significant agricultural research efforts are aimed at reducing the impact of the YDVs on the yield of the various crop systems by (i) altering planting time and/or pesticide application regimes to avoid the accumulation of high densities of aphids, (ii) tilling practices, and (iii) developing virus-resistance crop varieties (Chain et al., 2005; Royer et al., 2005; Kennedy and Connery, 2012).

The YDVs are members of the *Luteoviridae* family. All members of the *Luteoviridae* (luteovirids) have linear, positive-sense, 5.5–6 kb RNA genomes and are obligately aphid-transmitted in a circulative, persistent manner, with the exception of Pea enation mosaic virus 1 (PEMV1), which is mechanically transmissible in the presence of the umbravirus, PEMV2. The key genes conserved in all luteovirids are the major coat protein (CP) and readthrough domain (RTD) generated by translational readthrough of the CP open reading frame (ORF) stop codon, which provides a long carboxy-terminal extension to the CP (Brault et al., 1995; Brown et al., 1996). The CP and CP-RTD proteins provide the virion structure and aphid transmission properties, and they play a role in virus movement and tissue specificity within the plant (Brault et al., 1995; Chay et al., 1996a; Van Den Heuvel et al., 1997; Peter et al., 2009). ORF 4, which is embedded in the CP ORF but in a different reading frame, is also conserved in all luteovirids, except PEMV1. The product of ORF 4 (P4) has features of a cell-to-cell movement protein (Chay et al., 1996a; Schmitz et al., 1997), which may confer the property that all Luteoviridae except PEMV1 are confined to the phloem. Because the sequence encoding CP-RTD and P4 is the only part of the genome conserved in all luteovirids with the aforementioned exception of PEMV1, we call this region the *Luteoviridae* block (Miller et al., 2002).

*Luteoviridae* fall into three genera: *Luteovirus, Polerovirus*, and *Enamovirus* (Domier, 2012). Outside of the *Luteoviridae* block, the viral genomes are completely different between *Polerovirus* and *Luteovirus* genera. The RNA-dependent RNA polymerase (RdRp) genes (the key gene used for virus classification) of poleroviruses and the only enamovirus (PEMV1) are similar to each other but quite distantly related to those of genus *Luteovirus* (Miller et al., 2002; Domier, 2012). The polero/enamovirus RdRps are more similar to those of genus *Sobemovirus*, which has not been assigned to a family, than to those of genus *Luteovirus*. Moreover, outside of the *Luteoviridae* block, the genomes of genus *Luteovirus* (including the RdRp ORF) are most closely related to those of genus *Dianthovirus* in the *Tombusviridae* (Miller et al., 2002). In particular, the RdRp and translational control signals of genus *Luteovirus* resemble those of the *Tombusviridae* more than they resemble those of the *Polerovirus* or *Enamovirus* genera. Moreover, poleroviruses and the enamovirus contain a genome-linked protein (VPg), and also encode a viral suppressor of gene silencing (VSR) in ORF 0 (Pfeffer et al., 2002; Mangwende et al., 2009). Viruses in genus *Luteovirus*, like the *Tombusviridae*, have neither a VPg nor an ORF 0.

The original YDVs identified as the causal agents of yellow dwarf disease were all called BYDV and placed into five strains (now considered species) based on serotype, symptomatology and predominant aphid vector species (Rochow, 1969; Rochow and Muller, 1971). BYDV-RPV, -MAV, -SGV, and -RMV were found to be transmitted most efficiently by *R. padi, Sitobion avenae, Schizaphus graminum* and *R. maidis*, respectively. The most common virus is BYDV-PAV which is transmitted efficiently by *R. padi* and *S. avenae* (Rochow, 1969; Rochow and Muller, 1971).

Upon sequencing the complete genomes of some of the BYDV strains, it became clear that BYDV-RPV is a *Polerovirus*, renamed Cereal yellow dwarf (CYDV)-RPV, while BYDV-PAV and BYDV-MAV which have virtually identical RdRp sequences, are in genus *Luteovirus*. More recently discovered YDVs include CYDV-RPS, a CYDV-RPV-like virus that causes cork screw-shaped leaves and leaf notching in wheat; the former BYDV-GPV (now Wheat yellow dwarf virus-GPV (WYDV-GPV) (Zhang et al., 2009); and BYDV-PAS, a severe BYDV-PAV-like virus that breaks resistance in oat (Chay et al., 1996b).

In addition to the BYDVs, the genus *Luteovirus* includes Bean leafroll virus (BLRV), Soybean dwarf virus (SbDV), and (unofficially) Rose spring dwarf-associated virus (RSDaV). In addition to the CYDVs, the *Polerovirus* genus includes about two dozen viruses of diverse crops. Subsequently additional species have been identified in China, such as BYDV-GAV, and WYDV-GPV, transmitted primarily by *S. graminum* and *S. avenae*, and *S. graminum* and *R. padi*, respectively, and BYDV-PAV-CN which is transmitted efficiently by all three aphid species (Jin et al., 2004; Liu et al., 2007; Zhang et al., 2009). BYDV-GAV is very similar to BYDV-MAV (Jin et al., 2004; Zhang et al., 2009), WYDV-GPV is closely related to CYDV-RPV (Lucio-Zavaleta et al., 2001; Zhang et al., 2009), while BYDV-PAV-CN is highly diverged from other BYDVs (Liu et al., 2007). It should be noted that the vector specificity of the YDVs can vary by isolate within a virus species, by genotype of an aphid species, or under different environmental conditions (Lucio-Zavaleta et al., 2001). Thus, the YDVs are now classified by nucleotide sequence identity and genome organization, rather than by the most efficient aphid vector.

Until this report, the complete BYDV-RMV genome had not been sequenced, only the nucleotide region encoding the coat protein had been reported (Geske et al., 1996; Domier et al., 1997). RMV is the only BYDV transmitted efficiently by *R. maidis*, the corn leaf aphid. Hence it infects maize (Itnyre et al., 1999a,b). Moreover, maize serves as a reservoir for BYDV-RMV from which it can be transmitted to nearby wheat plots where stunting and yield losses ensue (Brown et al., 1984). BYDVs can reduce sweet corn yield dramatically by causing incomplete ear filling which can render entire harvests unmarketable (Beuve et al., 1999; Itnyre et al., 1999b). BYDV-RMV virions are difficult to purify, hence it has been little studied. Here, we report the first complete sequence of a BYDV-RMV genome. The genome organization and sequence of the RMV MTFE87 isolate indicates that BYDV-RMV is a member of genus *Polerovirus*, but it is not closely related to the CYDVs, WYDV or any other polerovirus. Therefore, we submit that BYDV-RMV is a unique species, with the proposed new name Maize yellow dwarf virus (MYDV).

## Materials and methods

### Biological characterization

The RMV MTFE87 isolate was obtained from Dr. T. W. Carroll, Montana State University in 1990. It was originally collected from an infected wheat plant growing on the Fort Ellis Experiment Station of Montana State University in 1987. The isolate has been continually propagated in Coast Black oats by regular transfer to new plants using *R. maidis.* Serological and aphid transmission properties of RMV MTFE87 were determined using a collection of antibodies to each of the YDV strains (Rochow and Carmichael, 1979; Webby and Lister, 1992). Double antibody sandwich (DAS) ELISA was carried out as described previously (Brumfield et al., 1992). Five different aphid species were used to determine the vector specificity of the RMV MTFE87 isolate. *R. maidis, R. padi, S. avenae, S. graminum* were previously described (Rochow and Carmichael, 1979; Power and Gray, 1995) and have been maintained as clonal lineages in the laboratory since their collection. The *Metapalophium dirhodum* colony was a gift from Fred Gildow (Gildow, 1993) and has been maintained in the laboratory in Ithaca, NY since 1993. Fourth instar or adult apterous aphids were allowed a 36–48 h acquisition access period on detached leaves of Coast Black oat plants infected with RMV MTFE87 4–5 weeks previously. Ten aphids were subsequently transferred to each of eight plants for a 72 h inoculation access period. Plants were fumigated and grown in a greenhouse and observed for symptoms for 3–4 weeks and tested using DAS-ELISA.

### Virus purification and viral RNA extraction

Virions were extracted from infected Coast Black oat plants as described previously (Hammond et al., 1983; Webby and Lister, 1992). Viral RNA was extracted using the hot phenol method. All centrifugation was at 13,200 rpm (16,100 × *g*) in a 24 × 1.5 ml tube rotor in an Eppendorf 5415R centrifuge. Briefly: purified virions were added to 3 volumes of extraction buffer yielding a final concentration of 167 mM Tris base (pH 8.5), 1% SDS, and 12.5 mM EDTA (all reagents from Sigma-Aldrich, St. Louis, MO). Hot (65°C) phenol was then added equal to the total volume, and after vortexing, the solution was incubated at 65°C for 15 min. The solution was vortexed again, centrifuged, and the aqueous phase saved. The phenol phase was back extracted using the above extraction buffer lacking SDS. After vortexing and centrifugation, the second aqueous phase was combined with the first. The combined aqueous phases were then extracted twice with equal volumes of phenol/chloroform/isoamyl alcohol (25:24:1). The RNA was precipitated at −20°C after adding 1/15 volume 3M sodium acetate (pH 5.5) and 2.5 volumes 95% ethanol. After centrifugation, the pellet was washed in 70% ethanol, vacuum dried and dissolved in a small volume of nuclease-free water.

### Amplification of genome downstream of the viral coat protein (CP)

The 3′ terminal region (1788 base pairs) of RMV MTFE87 was amplified and prepared for cloning using ligated-anchor PCR (LA-PCR) according to published protocols (Beckett and Miller, 2007). LA-PCR was performed on purified RMV MTFE87 viral RNA (500 ng). An anchor oligomer (5′-CTATAGTGTCACCTAAATGCGTGAAGAGCCTCCTACCAGCTGCTCCTATG-3′) was ligated directly to the 3′ end of the viral RNA. PCR amplification was conducted using an upstream primer (5′-AGATCACAAAAGTCATACTGGAGTTCATCT-3′) homologous to the viral coat protein (CP) and a downstream primer (5′-CATAGGAGCAGCTGGTAGGAGGCTCTTC-3′) complementary to the anchor.

### Amplification of genome upstream of the viral coat protein

Overlapping genomic sections extending from the viral CP to the 5′ end of the genome were amplified and prepared for cloning using the SMART™ RACE cDNA Amplification Kit (Clontech) according to manufacturer's instructions. The following viral-specific primers were used in conjunction with the kit to walk step-by-step to the 5′ end of the genome: 5′-ATGCGAGGGTGCTGAGCTTGTTGTG-3′, 5′-GGATGTCATCCTCATCATCAGCCCAGTTTC-3′, 5′-AGCCGGAGTTGGAAGCGTTTATAGC-3′.

### Cloning and sequencing of viral genome fragments

PCR amplified genome fragments were cloned using the Zero Blunt® TOPO® PCR Cloning Kit (Invitrogen) according to manufacturer's instructions. Chimeric plasmids were purified from positive transformants. The cloned viral fragments were sequenced either by primer-walking or by 96-well sequencing on an ABI 3730 × l DNA Analyzer at the ISU DNA Sequencing Facility. Plasmid template was transposon tagged in preparation for sequencing using the Template Generation System™ II (Finnzymes) according to manufacturer's instructions.

### Sequence assembly and analysis

The Phred, Phrap, and Consed software programs were used in tandem to process and assemble raw sequencing reads for all clones which were sequenced in a 96-well plate format (Ewing and Green, 1998; Gordon et al., 1998). Vector NTI® (Invitrogen) was used to assemble the sequence reads from primer-walked clones. The complete nucleotide sequence of the RMV MTFE87 genome, as well as the amino acid sequences of all six major proteins, were compared with a range of other fully sequenced luteovirids (Table 1). The sequences were organized within the JalView alignment editor, version 2.4 (Clamp et al., 2004). The full length genomes were aligned with Clustal W (Thompson et al., 1994). The amino acid sequences were aligned using the MUSCLE algorithm (Edgar, 2004). Phylogenetic relationships were inferred from these alignments using the Neighbor-Joining method with 1000 replicates for bootstrapping in the MEGA4 (Molecular Evolutionary Genetics Analysis version 4.0) software package (Tamura et al., 2007). Sequence identity was determined via the Needleman-Wunsch global alignment in the EMBOSS Pairwise Alignment Algorithms, http://www.ebi.ac.uk/Tools/emboss/align/index.html.

**Table 1:** **Virus abbreviations and GenBank accession numbers for viruses used for sequence comparisons in this study**.

**Virus name**	**Abbreviation**	**Accession Number**
Barley yellow dwarf virus—GAV	BYDV-GAV	NC_004666
Barley yellow dwarf virus—MAV	BYDV-MAV	NC_003680
Barley yellow dwarf virus—PAS	BYDV-PAS	NC_002160
Barley yellow dwarf virus—PAV	BYDV-PAV	NC_004750
Bean leafroll virus	BLRV	NC_003369
Beet chlorosis virus	BChV	NC_002766
Beet mild yellowing virus	BMYV	NC_003491
Beet western yellows virus	BWYV	NC_004756
Carrot red leaf virus	CtRLV	NC_006265
Cereal yellow dwarf virus—RPS	CYDV-RPS	NC_002198
Cereal yellow dwarf virus—RPV	CYDV-RPV	NC_004751
Chickpea chlorotic stunt virus	ChCSV	NC_008249
Cucurbit aphid-borne yellows virus	CABYV	NC_003688
Melon aphid-borne yellows virus	MABYV	NC_010809
Pea enation mosaic virus 1	PEMV1	NC_003629
Potato leafroll virus	PLRV	NC_001747
Barley yellow dwarf virus-RMV (proposed new name: Maize yellow dwarf virus-RMV)	BYDV-RMV (MYDV-RMV)	KC921392
Rose spring dwarf-associated virus	RSDaV	NC_010806
Soybean dwarf virus	SbDV	NC_003056
Sugarcane yellow leaf virus	ScYLV	NC_000874
Tobacco vein distorting virus	TVDV	NC_010732
Turnip yellows virus	TuYV	NC_003431
Wheat yellow dwarf virus—GPV	WYDV-GPV	NC_012931

## Results

### Biological characterization of the RMV MTFE87 isolate

BYDV-RMV MTFE87 reacted in DAS-ELISA with antibodies to BYDV-RMV, but not to antibodies made against the other viruses (BYDV-PAV, BYDV-MAV, BYDV-SGV or CYDV-RPV). RMV MTFE87 was transmitted efficiently by *R. maidis* to eight of eight plants, to a lesser extent by *S. graminum* (4/8) and *R. padi* (1/8) and was not transmitted by *Metapalophium dirhodum* or *S. avenae* (both 0/8) FE87 was passaged six times in Coast Black oats using 10 *R. maidis* or *S. graminum* apterous adults for each passage. Transmission efficiency remained at 100% for *R. maidis* and increased to 95% for *S. graminum* by the sixth serial passage. The virus continued to react only with anti-BYDV-RMV antibodies following all of the serial passages by either aphid. Symptoms induced by RMV MTFE87 isolate were more severe on oat and wheat than the type RMV isolate from NY (RMV-NY) (Rochow and Norman, 1961). The RMV-NY isolate rarely induces visible symptoms in wheat and many cultivars of oat. Symptoms in Coast Black oat are mild chlorosis or reddening of mature leaf tips, whereas the RMV MTFE87 isolate induced yellowing or reddening of flag leaves in wheat, noticeable stunting of the plants and incomplete filling of heads. Oat plants infected with RMV MTFE87 were severely stunted, with reddening and necrosis of leaves and incomplete formation of seed. The severe symptoms in oat and wheat are typical of RMV isolates collected in Montana (Brumfield et al., 1992) in contrast to RMV isolates collected in NY (Lucio-Zavaleta et al., 2001).

### Genome organization of BYDV-RMV

**Figure 1:**
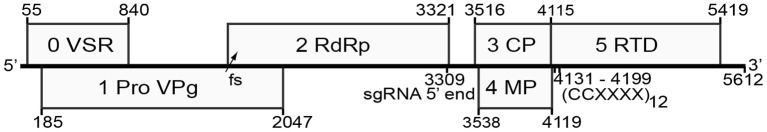
**Genome organization of BYDV-RMV.** Numbers in small font indicate genomic positions of each ORF (numbered in large font) and the position of the predicted subgenomic RNA 5′ end and the readthrough sequence. VPg, viral genome-linked protein; VSR, putative viral suppressor of RNA silencing; RdRp, RNA-dependent RNA polymerase; CP, coat protein; MP, movement protein; RTD, readthrough domain; fs, site of −1 ribosomal frameshift; sgRNA 5′ end, predicted 5′ end of subgenomic RNA1 at nt 3309; (CCXXXX)_12_, repeat motif required for readthrough of the CP ORF stop codon at nts 4131–4199.

The nucleotide sequence of RMV MTFE87 genomic RNA was found to be 5612 nt long, encoding six ORFs (Figure 1). The 5′ and 3′ untranslated regions (UTRs) are 54 and 158 nt long, respectively, and the only intergenic region spans nucleotides 3322–3515. The arrangement and sequences of ORFs resemble those of poleroviruses (Figure 1, Table 2). Based on sequence comparisons with poleroviruses, the ORFs encode a putative viral suppressor of silencing (VSR, ORF 0), serine protease and VPg (both in ORF 1), RdRp (ORF 2), coat protein (ORF 3), putative movement protein (ORF 4, which overlaps with ORF 3), and the CP readthrough domain (ORF 5) (Figure 1). A feature found in only three other luteovirids—Chickpea chlorotic stunt virus (ChCSV), Melon aphid-borne yellows virus (MABYV), and Cucurbit aphid-borne yellows virus (CABYV), is that ORF 4 of BYDV-RMV extends beyond the end of ORF 3, in this case by 4 nt. In all other *Luteoviridae*, the stop codon of ORF 4 is upstream of the CP ORF stop codon.

**Table 2:** **Sequence identity (%)^a^ of BYDV-RMV proteins to those of other luteovirids**.

**Genus**	**Virus^a^**	**P0**	**P1**	**P2**	**P3**	**P4**	**P5**
*Luteovirus*	BLRV	NA^b^	7	14	51	31	26
*Luteovirus*	BYDV-GAV	NA^b^	10	16	45	28	31
*Luteovirus*	BYDV-MAV	NA^b^	9	15	45	26	29
*Luteovirus*	BYDV-PAS	NA^b^	7	14	42	19	31
*Luteovirus*	BYDV-PAV	NA^b^	10	15	45	21	31
*Luteovirus*	RSDaV	NA^b^	2	19	32	25	31
*Luteovirus*	SbDV	NA^b^	7	14	51	38	26
*Polerovirus*	BChV	15	27	57	60	37	25
*Polerovirus*	BMYV	20	33	59	60	39	25
*Polerovirus*	BWYV	22	31	57	61	38	24
*Polerovirus*	CABYV	22	34	57	59	43	33
*Polerovirus*	ChCSV	18	30	60	59	37	29
*Polerovirus*	CtRLV	17	32	62	48	28	30
*Polerovirus*	CYDV-RPS	19	30	52	58	32	28
*Polerovirus*	CYDV-RPV	20	29	51	61	33	27
*Polerovirus*	MABYV	23	33	58	55	41	31
*Polerovirus*	PLRV	11	30	56	55	35	25
*Polerovirus*	ScYLV	18	30	56	40	29	37
*Polerovirus*	TuYV	21	39	64	61	38	26
*Polerovirus*	TVDV	23	32	62	53	31	26
*Polerovirus*	WYDV-GPV	20	30	53	59	35	27
*Enamovirus*	PEMV1	23	18	37	30	NA^b^	30

a The identity of the sequences to BYDV-RMV was determined with the EMBOSS Needle global pairwise alignment algorithm. Molecular weights for the BYDV-RMV proteins were estimated using the ExPASy Server (Gasteiger et al., 2003).

b NA, not applicable.

### Whole genome alignments of *Luteoviridae*

**Figure 2:**
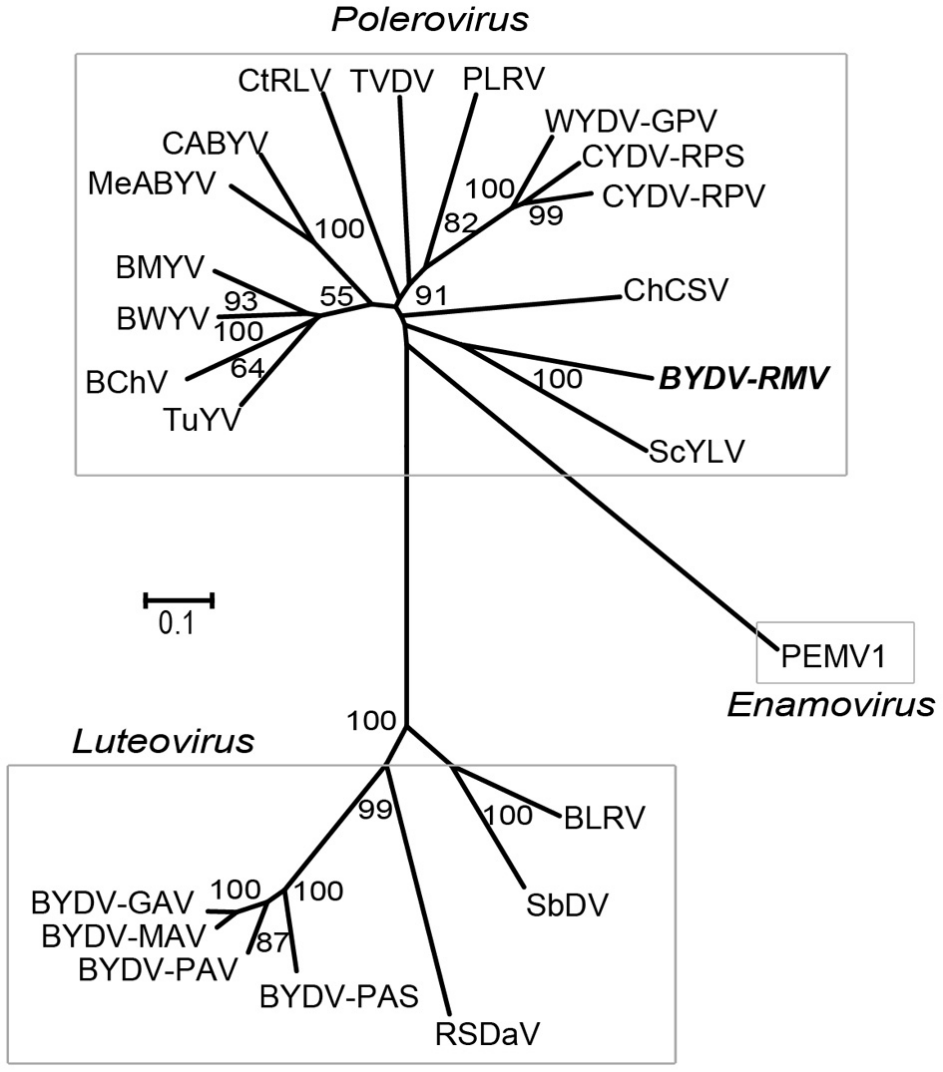
**Neighbor-joining tree of Clustal W-aligned whole genome sequences of luteovirids generated in the MEGA 4 software package.** Numbers representing bootstrap values when greater than 50% for 1000 replicates are shown.

The full-length luteovirid genomes found in GenBank REFSEQ database were aligned by the neighbor-joining method using MEGA4 (Tamura et al., 2007) with 1000 replications (Figure 2). BYDV-RMV was grouped with the *Polerovirus* genus and in 100% of the replicates was closest to, but highly distinct from, Sugarcane yellow leaf virus (ScYLV). The BYDV-RMV/ScYLV branch was separated deeply from the CYDV and WYDV grouping indicating that BYDV-RMV is more closely related to other poleroviruses than it is to CYDVs and WYDV. As expected in this comparison, genus *Luteovirus* was well-separated from the *Polerovirus* and *Enamovirus* groups.

### Protein alignments and analyses of RMV MTFE87 and selected luteovirids

**Figure 3:**
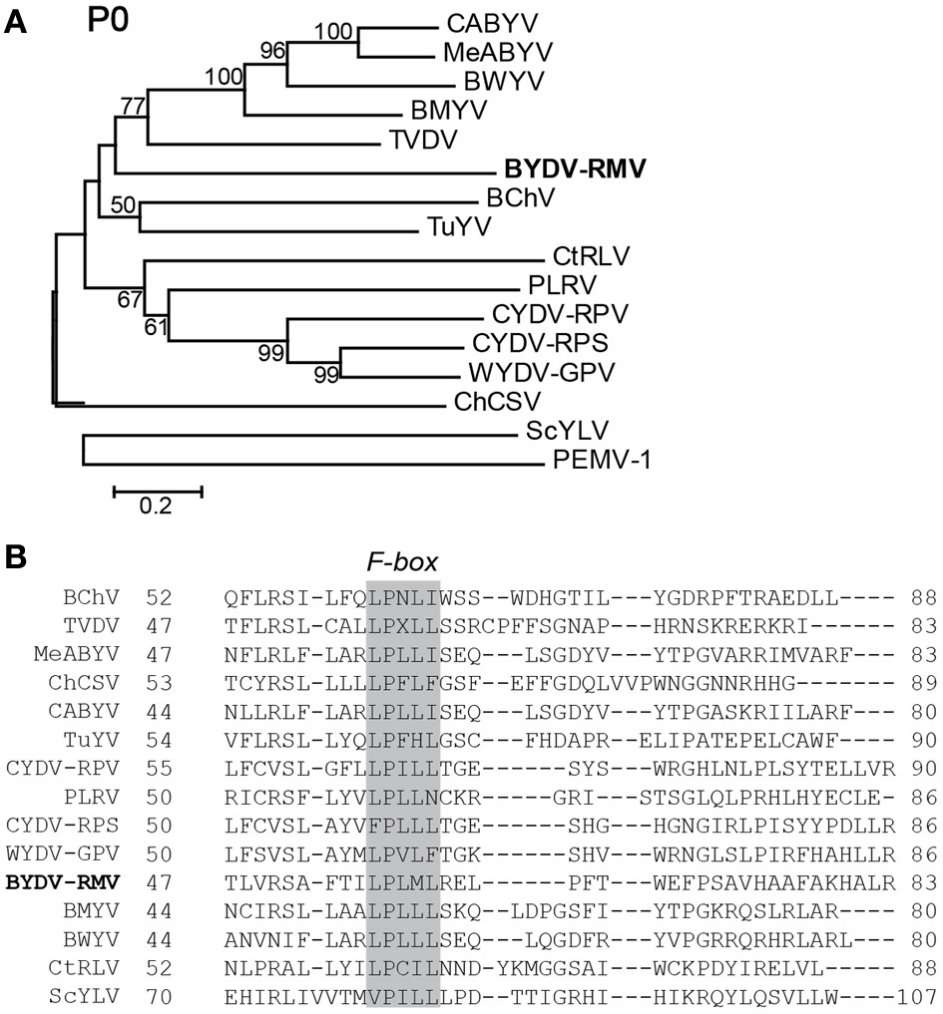
**Analysis of P0 amino acid sequences. (A)** Amino acid sequences of the P0s for viruses in the *Polereovirus* and *Enamovirus* genera were aligned with the MUSCLE algorithm within JalView. The aligned sequences were used for inferring evolutionary relationships using the Neighbor-Joining method. The resultant tree, drawn to scale, is shown with the percentage (greater than 50% only) of 1000 bootstrap replicates. **(B)** A portion of the MUSCLE aligned P0 showing the conserved F-box domain LPxxL/I among the *Polerovirus* members. Accession numbers of viral genome sequences used in the alignment are in Table 1.

The amino acid sequences of selected polerovirus and enamovirus proteins were aligned using the MUSCLE (Edgar, 2004) algorithm and from these alignments we created neighbor-joining trees via the MEGA4 software package (Tamura et al., 2007). P0 of RMV MTFE87 is separated readily from the CYDV/WYDV group and is most closely related to the Beet chlorosis virus (BChV)/Turnip yellows virus (TuYV) branch (Figure 3A). The CYDV-RPS, CYDV-RPV, and WYDV group separates from the remainder of the sequences in 99% of the bootstrap replicates.

**Figure 4:**
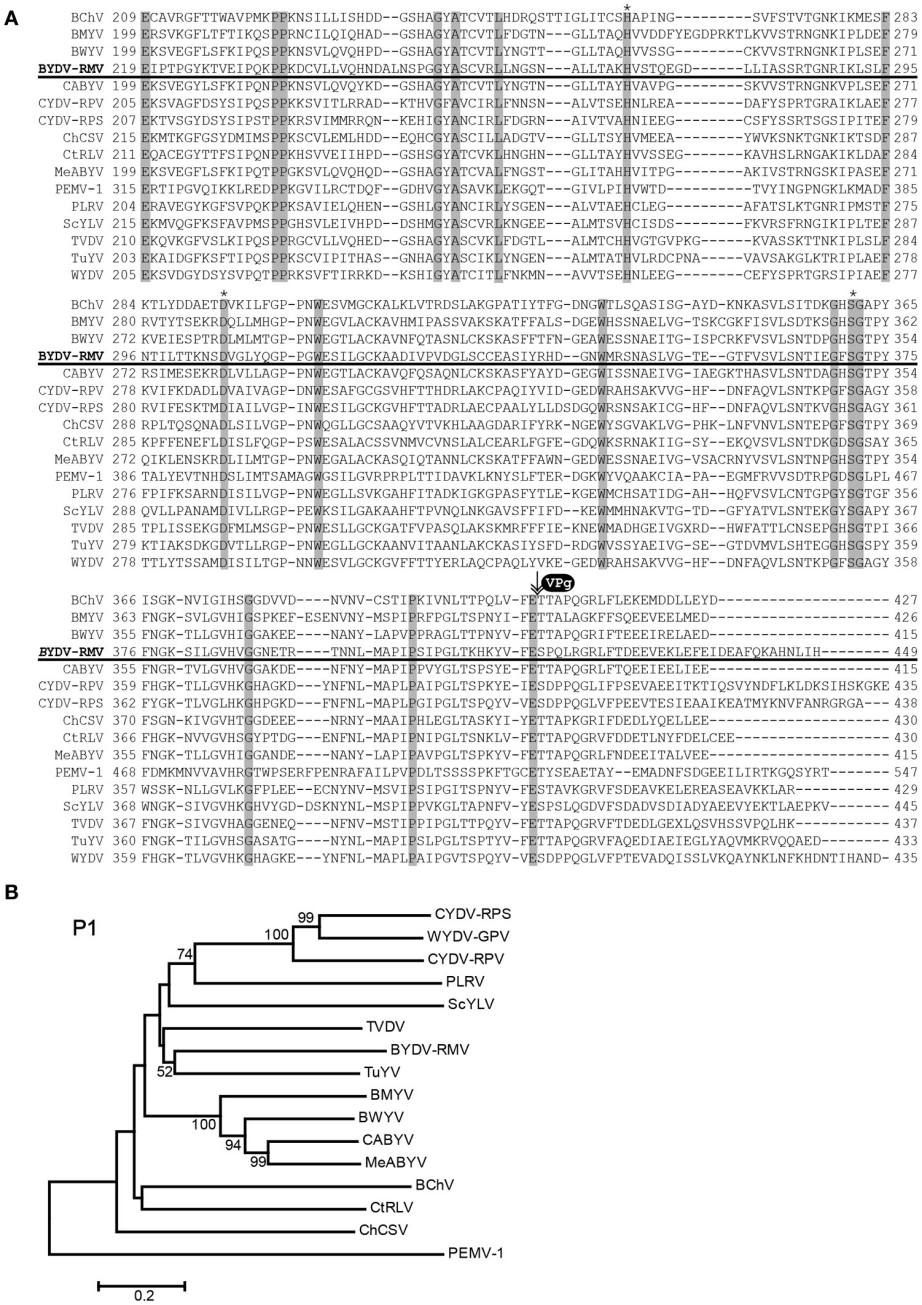
**Analysis of P1 amino acid sequences. (A)** Amino acid sequences of P1 were aligned with the MUSCLE algorithm within JalView. The region with the protease domain is shown. Shaded boxes indicated conserved amino acids. Asterisks indicate amino acids of the catalytic triad in the protease active site. VPg and arrow indicate predicted proteolytic cleavage site that forms the C-terminus of the protease and the N-terminus of the VPg. **(B)** The MUSCLE aligned P1s for virus in the *Polerovirus* and *Enamovirus* genera were used for inferring evolutionary relationships using the Neighbor-Joining method. The resultant tree, drawn to scale, is shown with the percentage (greater than 50% only) of 1000 bootstrap replicates.

P1 of poleroviruses is a polyprotein that is cleaved by its internal protease into functional polypeptides that include the N terminus, the protease, the VPg, and a downstream RNA-binding fragment of unknown function (Prüfer et al., 1999, 2006; Li et al., 2000). The key amino acids of the catalytic triad in the protease (Li et al., 2000) are at positions 272, 306 and 373 (Figure 4A). Based on the known N-termini of the Potato leafroll virus (PLRV) VPg (Van Der Wilk et al., 1997b) and the enamovirus PEMV1 (Wobus et al., 1998), the N-terminus of the VPg of RMV MTFE87 is predicted to be amino acid T417 (Figure 4A). RMV MTFE87 P1 is most closely related to P1 of TuYV and TVDV, distinct from the CYDV/WYDV group linked in 100% of the bootstrap replicates (Figure 4B).

**Figure 5:**
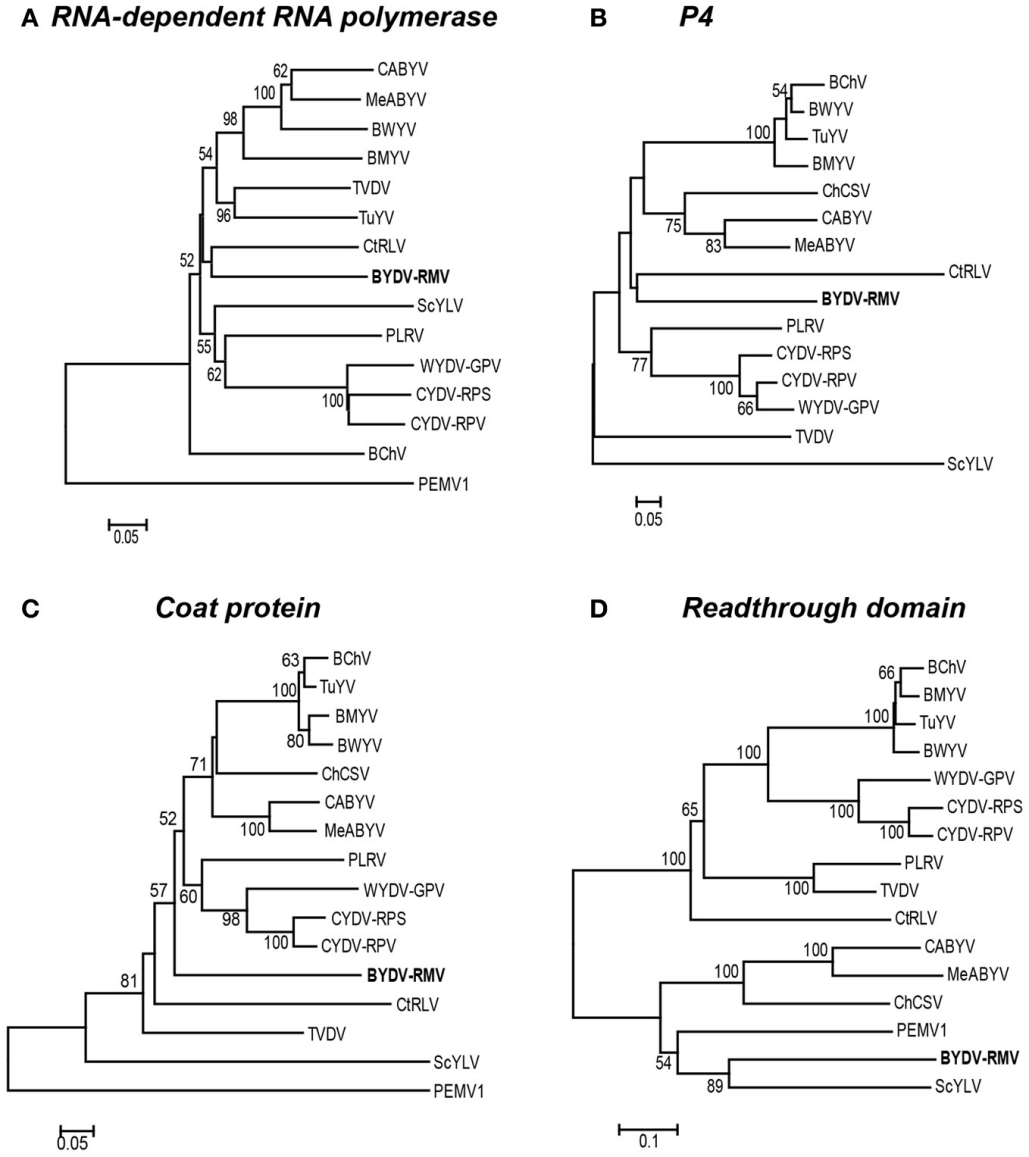
**Phylogenetic relationships of four luteoviral proteins.** For each protein, the amino acid sequences were aligned using the MUSCLE algorithm in the JalView program and subsequently used to generate a Neighbor-Joining tree with the percentage of 1000 bootstrap replicates reported on a drawn-to-scale tree. Phylogenetic trees of: **(A)** RNA-dependent RNA polymerase (P2), **(B)** putative movement protein of P4, **(C)** the major coat protein (P3), and **(D)** the readthrough domain (P5) are shown.

In all studied poleroviruses, P2, which encodes the active site of the RdRp, is expressed only as a fusion with P1, as it is translated by frameshifting of ribosomes from ORF 1 to ORF 2 in the region of overlap (Prüfer et al., 1992; Kujawa et al., 1993; Miller and Giedroc, 2010). The CYDV/WYDV RdRp (P2) sequences clustered tightly in 100% of the replicates, but they all were strikingly distinct from RMV MTFE87 P2, which groups rather distantly with CtRLV (Figure 5A).

The relationship of the RMV MTFE87 CP to that of other poleroviruses is not well resolved. The CP sequences of PEMV1 (*Enamovirus*), and ScYLV are distinct from all of the remaining *Polerovirus* members, while 57 and 52% of bootstrap replicates support BYDV-RMV separation from TVDV and the large group of the remaining poleroviruses, respectively. But the close relatedness of the CYDV/WYDV CP to each other is present in 98% of the bootstrap replicates (Figure 5C).

The P4 proteins of TVDV and ScYLV are discretely separated from the remaining poleroviruses. BYDV-RMV and CtRLV form an intermediate between two larger clusters, one housing PLRV and CYDV/WYDV group while the other contains all others used in our study (Figure 5B). The readthrough domain, P5, is expressed as a fusion with the CP by leaky scanning through the amber stop codon of P3 (Brault et al., 1995; Brown et al., 1996). For the RTD, the *Polerovirus* and *Enamovirus* members branch into two distinct clades. RMV MTFE87 P5 groups with Sugarcane yellow leaf virus (ScYLV), CABYV, MABYV, ChCSV and PEMV1, in one clade. The remaining poleroviruses are in the other major clade including the CYDV/WYDV group present in 100% of bootstrap replicates (Figure 5D).

## Discussion

### Proteins of the *Luteoviridae*

P0 of RMV MTFE87 is a putative viral suppressor of RNA silencing (VSR) because it shares homology with other poleroviruses in which P0 is a VSR. P0 of TuYV (formerly Beet western yellows virus-FL, BWYV-FL), PLRV and PEMV1 has been shown to suppress the host plant's defensive posttranscriptional gene silencing (PTGS) system (Pfeffer et al., 2002; Mangwende et al., 2009; Fusaro et al., 2012) by inducing the host to degrade the key *Argonaute 1* (AGO1) protein (Baumberger et al., 2007; Bortolamiol et al., 2007). The F-box domain, LPxxL/I, which is conserved in the otherwise highly variable P0 of all luteovirids including BYDV-RMV (Figure 3B), is required for VSR activity, as it recruits proteins to form the E3 ubiquitin ligase activity that ubiquitylates AGO1, which leads to its degradation (Pazhouhandeh et al., 2006; Bortolamiol et al., 2007) by the autophagy pathway (Derrien et al., 2012). Moreover, transgenic expression of ORF0 of PLRV in potatoes, but not *Nicotiana*, is sufficient to produce virus-like symptoms (Van Der Wilk et al., 1997a). However, not all P0 proteins show VSR activity, for example those of Beet chlorosis virus (BChV) and some strains of Beet mild yellowing virus (BMYV) (Kozlowska-Makulska et al., 2010) and CYDV-RPV (Véronique Ziegler-Graff, personal communication) do not display VSR activity in standard assays. Thus, VSR function may vary depending on the virus strain–host species interaction.

The putative movement proteins of BYDV (Chay et al., 1996a,b) and PLRV (Lee et al., 2002) encoded by ORF 4 have been shown to allow host-dependent movement of the virus throughout the plant. P4 of BYDV-GAV was reported to contain an RNA-binding motif at its C-terminus with four arginine residues (Xia et al., 2008). The P4 proteins of other luteovirids were also shown to contain multiple arginine residues. In line with these observations, there are four arginine residues within the C-terminal 11 amino acids of the RMV P4 sequence.

### Modular evolution of *Luteoviridae*

As has been apparent since the first luteoviruses were sequenced, it is clear that luteovirid genes evolve at different rates. Note the striking lack of sequence homology among the P0 proteins (other than the F-box motif) which have around 15–23% homology to that of RMV MTFE87 (Table 2). This high sequence divergence of VSRs relative to other ORFs in related viruses occurs in other virus families with VSRs that act by entirely different mechanisms (Nayak et al., 2010). We speculate that VSRs are at the forefront of the evolutionary back-and-forth between virus and host immune system, which leads to rapid change, as the VSR must constantly out-evolve the host's defenses. In contrast, P2 is more conserved with 51–62% sequence identity among all of the members of the genus *Polerovirus*. The CPs of other poleroviruses have 40–60% sequence identity to the RMV MTFE87 CP, while the overlapping P4s have significantly less similarity (Table 2). This suggests that base changes in the ORF 3/4 sequence that alter the meaning of ORF 4 codons are more often tolerated than those that alter the amino acid sequence of the CP. Overall, the lack of high similarity of any luteovirid ORF with those of RMV MTFE87 emphasizes its uniqueness as a virus.

### CIS-acting signals

Cis acting sequences required for polerovirus translation, subgenomic mRNA (sgRNA) transcription and RNA synthesis have been identified in PLRV and others. The genomes of all poleroviruses begins with the sequence ACAAAA. Similarly, where known, the 5′ end of the sgRNA of the poleroviruses begins with ACAAAA (Miller and Mayo, 1991). This leads us to predict that the sgRNA required for translation of BYDV-RMV ORFs 3, 4, and 5 begins at position _3309_ACAAAA_3314_. This is 202 nt upstream of the CP ORF that starts at position 3516, giving an sgRNA leader sequence similar to the 212 nt leader of PLRV sgRNA1.

**Figure 6:**
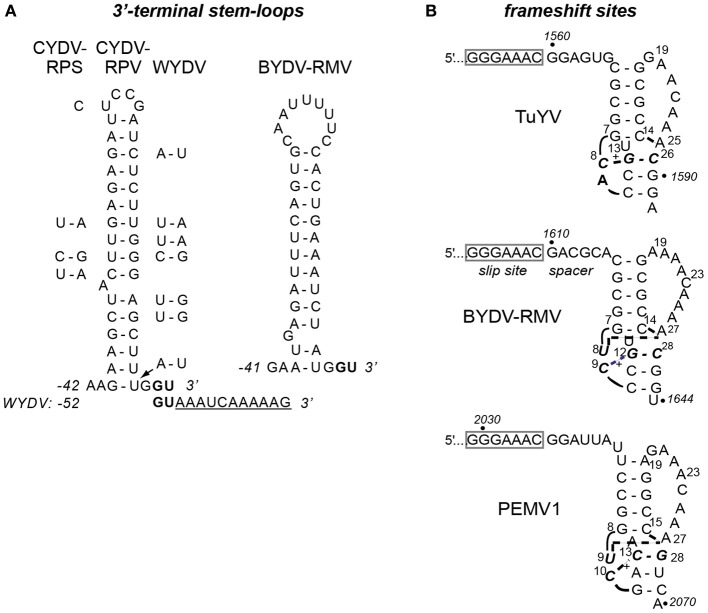
**Predicted secondary structures in BYDV-RMV RNA. (A)** Stem-loop at the 3′ terminus of CYDV-RPV genome as determined by Osman et al. (2006), flanked by base differences in CYDV-RPS (left) and WYDV (right) which show covariations in base pairing that maintain secondary structure. Conserved 3′ terminal bases, GU are shown in bold. Eleven extra bases at the 3′ end of the WYDV genome, not present in any other polerovirus, are shown below the CYDV-RPV sequence in underlined text. The proposed secondary structure of the 3′ end of the BYDV-RMV genome was predicted using Mfold (Zuker, 2003). Base numbering (negative) is from the 3′ end of the genomes. **(B)** Predicted (BYDV-RMV) and known (PEMV1 and TuYV) (Su et al., 1999; Nixon et al., 2002; Miller and Giedroc, 2010) tertiary structures of pseudoknots downstream of the frameshift sites (boxed). Italics indicate base numbers in the genome. Other numbering is the position in the fragment used for nmr (except BYDV-RMV where the number allows comparison with the other structures). Short curved lines indicate phosphodiester backbone as necessary for two-dimensional rendering. Bold, dashed lines indicate non-Watson-Crick interactions between bases. ^+^indicated protonated cytidine that participates in base triples. Due to the recent change of the name of the BWYV isolate used in previous structural studies (Domier, 2012) it is now indicated by the new name, TuYV.

A 40 nt stem-loop containing a bulged adenosine, ending 3 nt upstream of the 3′ end of the genome, was shown to be required for initiation of CYDV-RPV negative strand synthesis (Osman et al., 2006). This stem-loop is conserved in CYDV-RPS and WYDV, which are very closely related to CYDV-RPV (Figure 6A). In contrast, RMV MTFE87 has a different predicted stem-loop that is 39 nt long with a much larger loop and a bulged guanosine (Figure 5A). Like all other poleroviruses (except WYDV), the two bases at the 3′ end of the RMV MTFE87 genome are GU (bold, Figure 6A). Thus, the first two bases incorporated by the RdRp initiating synthesis of either strand are AC. Surprisingly, WYDV is reported to contain an 11 nt A-rich tract at the 3′ end downstream of the GU (Figure 6A). We speculate that this is either sequence added during 3′ RACE, or sequence of a defective WYDV genome. The distinct 3′ stem-loop of RMV MTFE87 further supports that above phylogenetic comparisons about the un-CYDV-like nature of BYDV-RMV.

We also identified probable translational control sequences. In all *Luteoviridae*, ORF2 encoding the active site of the RdRp is translated via ribosomal frameshifting at a shifty heptanucleotide, fitting the motif XXXYYYZ (where X is any base, Y is A or U, and Z is any base except G), in the region of ORF1–ORF2 overlap. Seven nt downstream of this site, the polero- and enamovirus genomes fold into a small, compact pseudoknot that pauses the ribosome to facilitate frameshifting (Su et al., 1999; Nixon et al., 2002; Miller and Giedroc, 2010). Indeed, in the region of ORF1–ORF2 overlap in the RMV MTFE87 genome, we found the shifty heptanucleotide _1603_GGGAAAC_1609_, followed by a predicted pseudoknot that spans bases 1616–1643 (Figure 6B). This pseudoknot closely resembles those of other poleroviruses and the enamovirus, the structures of which have been determined at high resolution by NMR (Su et al., 1999; Nixon et al., 2002). The Watson–Crick helices of the RMV MTFE87 pseudoknot, CGCGG:CCGCG and CCG:CGG are identical to those in TuYV, but the helical junction region includes a predicted C+-AU triplet that resembles the junction in the PEMV1 pseudoknot (Figure 6B).

The sequence required for translational readthrough of the CP ORF stop codon also resembles those of other *Luteoviridae*. Readthrough of the BYDV-PAV CP ORF stop codon was shown to require at least five repeats of the sequence CCXXXX, where X is any base, beginning about 16–22 nt downstream of the stop codon (Brown et al., 1996). Indeed, in the RMV MTFE87 genome, a tract of 12 CCXXXX repeats starting at nt 4131 begins 16 nt downstream of the CP ORF stop codon (Figure 1). This codes for an amino acid sequence of alternating proline residues, which is a likely spacer to permit separate folding of the CP and RTD protein domains. In summary, BYDV-RMV has all the known cis-acting signals of a polerovirus to control translation of viral proteins.

### Proposed name change of BYDV-RMV to maize yellow dwarf virus-RMV

The RMV MTFE87 sequence shows that the viruses once called BYDV are even more diverse than previously thought. The sequence also shows clearly that BYDV-RMV is not in genus *Luteovirus*, to which all other sequenced viruses currently called BYDV are assigned. Nor is it a type of CYDV or WYDV. Therefore, we propose to change the name of BYDV-RMV to Maize yellow dwarf virus-RMV (MYDV-RMV) (Miller et al., 2013). This name (1) acknowledges that the virus is clearly a new species (2) is consistent with observations that BYDV-RMV often infects maize (Brown et al., 1984; Beuve et al., 1999; Itnyre et al., 1999a,b), (3) retains the RMV notation for the predominant vector, *R. maidis* (although *S. graminum* and *R. padi* can also be efficient vectors, particularly in the western United States), and (4) retains the YDV descriptor long used for luteovirids that infect cereals.

### Conflict of interest statement

The authors declare that the research was conducted in the absence of any commercial or financial relationships that could be construed as a potential conflict of interest.
